# Intraoperative Doppler Ultrasound for Detection of Early Postoperative Vascular Complications in Orthotopic Liver Transplants

**DOI:** 10.7759/cureus.26077

**Published:** 2022-06-19

**Authors:** Raymond I Okeke, Jeffery Bettag, Reeder Wells, Michaela Wycoff, Taylor Hallcox, Justin Lok, Alexandra Phocas, David L Annakie, Ramy Shoela, Mustafa Nazzal

**Affiliations:** 1 Surgery, Saint Louis University Hospital, St. Louis, USA; 2 Medicine, Saint Louis University School of Medicine, St. Louis, USA; 3 Radiology, Saint Louis University Hospital, St. Louis, USA

**Keywords:** intraoperative, orthotopic liver transplant, vascular complication, intraoperative ultrasounds, doppler ultrasound

## Abstract

Liver transplantation is currently the only curative treatment for patients with end-stage liver disease. However, liver transplantation can be associated with catastrophic complications in the early postoperative setting, including hepatic artery thrombosis (HAT) and portal vein thrombosis (PVT). Postoperative complications are associated with hepatic artery resistive index (RI) < 6, systolic acceleration time (SAT) > 0.08 seconds and peak systolic velocity (PSV) > 200 cm/s on doppler ultrasound (DUS). DUS is also used in an intraoperative setting to assess patency and early complications prior to the end of the operative period, allowing for early correction. This literature review evaluates the prevalence of DUS use in intraoperative settings to identify transplant complications. A lack of consistency and minimal knowledge of intraoperative DUS warrants additional research into its usage and standardization.

## Introduction and background

A liver transplant (LT) is the only curative treatment for children and adults with end-stage liver disease. The first LT performed by Starzl in 1967 was from a deceased donor. A whole donor liver replaced the resected liver and was called an orthotopic liver transplant (OLT) [1,2]. The introduction of cyclosporine in 1979 by Calne improved graft rejection by immunosuppression [3]. With better immunosuppression, vascular complications are now the most common cause of morbidity and mortality following LT [4]. Doppler ultrasonography (DUS) is the current modality of choice in monitoring postoperative vascular complications. It is a non-invasive and cost-efficient technique for determining adequate perfusion and outflow of the graft [5,6]. The use of DUS to detect postoperative vascular complications following LT is well-documented. Ultrasound (US) is the first-line imaging modality used in evaluating early and late complications following transplantation [7-9]. It makes an actionable diagnosis or prompts more invasive imaging such as computed tomography angiography (CTA) or magnetic resonance (MR) cholangiography. DUS assessment has been expanding to all stages of liver transplant evaluation. This literature review aimed to assess the utility of perioperative DUS parameters in identifying and preventing complications.

## Review

General considerations in liver transplant ultrasound 

The standard technique for perioperative Doppler ultrasound (DUS) involves using a 2-5 MHz convex transducer positioned with a probe angle < 60 degrees to the long axis of the vessel. Typically, hepatic artery measurements are made just proximal to the hepatic artery bifurcation [10-15]. The arterial and biliary anastomoses are studied with similar instrumentation and technique as described above. DUS assessment of the liver and its vasculature is affected by excess probe pressure, the respiratory cycle, and GI transit. Therefore, postoperative DUS assessment can be limited in patients who are obese, cannot control breathing, or have not fasted for 4-6 hours [16]. However, these factors are less concerning in intraoperative DUS, where probes can be placed directly on the graft and its vasculature.

Postoperative Doppler ultrasound

In 1994, Dodd et al. were among the first to associate quantitative DUS findings in the postoperative period with hepatic arterial complications following a liver transplant. Their findings from a retrospective cohort identified a significant decrease in the hepatic arterial resistive index (RI) and a significant increase in systolic acceleration time (SAT) in patients that experienced arterial complications (thrombosis or stenosis). Peak systolic velocity (PSV) and absent arterial waveforms were not associated with vascular complications. The results suggested that a RI < 0.5 and SAT > 0.08 seconds were predictive of hepatic arterial complications [17]. These findings set early thresholds for postoperative DUS screening in liver transplants. Almost 10 years later, Vit et al. performed a similar investigation in a similar-sized cohort and found SAT > 0.08 seconds, but not RI, to predict arterial complications after transplant [15]. Dodd et al. and Vit et al. agreed that PSV was not a useful parameter for detecting hepatic artery stenosis (HAS) and hepatic artery thrombosis (HAT) [15,17]. The accepted normal reference ranges for DUS parameters following liver transplant are hepatic artery RI > 0.5 (with normalization to <0.8 within 72 hours), SAT < 0.08 seconds, and hepatic artery PSV < 200 cm/s at the anastomosis [11]. 

The incidence of early hepatic artery thrombosis (eHAT), defined as hepatic artery thrombosis (HAT) within one month of transplant, is a well-documented complication following both OLT and SLT. eHAT occurs in 2-5% of adults following LT [18-20]. A retrospective study by Wu et al. looking at adult OLT patients found that eHAT occurred at a mean of ten days after transplant with mortality of almost 50% (6/14) [18]. The early identification and treatment of HAT prevent further complications such as biliary stricture and biliary leak, as the biliary tree is supplied exclusively by the hepatic artery and its branches [19-21]. Garcia-Criado et al. found that approximately half of liver transplant patients demonstrate a transient increase in hepatic artery RI within the first 72 hours postoperatively [12]. Garcia-Criado et al., in 2009, showed that ultrasound diagnosis of HAT is made either at the hilum or at the intrahepatic hepatic arteries [22]. Uzochukwu et al. found that main, left, and right hepatic artery RIs < 0.6 in a cohort of OLT patients were associated with an increased incidence of graft complications requiring intervention in the early postoperative period [23]. Kimura et al. also concluded that decreasing diastolic flow and peak systolic velocity over time were predictors of imminent HAT [11]. Their findings contrast with Dodd et al., who found RI and SAT as good parameters in identifying hepatic artery compromise [17]. A retrospective study by Tezcan et al. found that portal venous flow significantly decreased from 70 cm/s to 52 cm/s within 1 hour after successful treatment of HAT, suggesting that compensatory portal venous flow due to compromised arterial flow is a potential alternative indicator of HAT [14].

Hepatic artery stenosis (HAS) occurs later than HAT postoperatively [24]. It occurs secondary to clamp injury or vasa vasorum disruption [25]. The incidence of post-transplant HAS is 5-13% [24]. It is associated with increased morbidity, decreased patient survival, and non-anastomotic biliary strictures [24,25]. DUS is the first-line imaging modality for HAS. Findings distal to the site of stenosis include an increase in diastolic flow, a decrease in RI to less than 0.5-0.55, and an increased SAT > 0.08 seconds. There is also a characteristic tardus et parvus spectral waveform. DUS identifies hemodynamically significant stenosis at 70-83% sensitivity and 60-73% specificity [8]. A retrospective study by Platt et al. similarly reported that decreased hepatic artery RI and increased arterial SAT were independent predictors of HAS in the postoperative period. When combined, RI and SAT had a specificity of 96% and a sensitivity of 67% [26]. The lower sensitivity of this combined parameter is not ideal for screening for HAS in the postoperative period. Lall et al. followed a cohort of OLT patients with HAS treated with stenting and reported that a pre-stenting RI < 0.4 in the main hepatic artery was predictive of restenosis with a sensitivity of 100%. A poststenting PSV > 300 cm/s was predictive of restenosis with high sensitivity when assessed more than 90 days after stenting. At least three days after stenting, RIs <0.55 in 3 or more hepatic arterial locations had 100% sensitivity for restenosis with increased specificity up to 70.5%, compared to RI <0.55 in one or two arterial locations [27]. In contrast, a more extensive cohort study by Mohamed et al. found hepatic artery PSVs to have little diagnostic value for HAS, while intrahepatic hepatic artery RI < 0.585 and SAT > 0.045 seconds were predictive with sensitivities and specificities >80% for HAS in the early postoperative period [28]. Decreased hepatic artery RI should raise concern for HAS. RI assessment using DUS is an excellent screening tool in the days to months following transplant. Ultrasound can identify associated biliary strictures that often coincide with HAS. Liao et al. reported that assessment of the bile duct with ultrasound (US) at the hilum showing a diminished or absent bile duct lumen had a sensitivity of 94% and a specificity of 84% for non-anastomotic stricture when compared to cholangiography. Many cases of non-anastomotic stricture and anastomotic strictures are from concomitant HAS [29].

Intraoperative Doppler ultrasound

Cheng et al. demonstrated the utility of intraoperative DUS in a case series of nine patients with identified vascular complications. The subsequent intervention resulted in surgical correction at the time of transplant and 100% graft survival [30]. Gu et al. investigated more quantitative assessments in pediatric SLTs and found that hepatic artery diameters < 2 mm, hepatic artery PSV < 40 cm/s, and hepatic artery RI < 0.6 intraoperatively were predictive of eHAT [31]. Hepatic artery RI < 0.6 was the most predictive, with a sensitivity of 86% and specificity of 89% [31]. Choi et al. ran a retrospective study with standard intraoperative DUS parameters in adult and pediatric SLT patients. They determined that hepatic artery (SAT) > 0.08 seconds and the presence of tardus-parvus wave pattern are more predictive for HAS or HAT than RI< 0.6 or PSV increase >200 cm/s [13]. However, the sensitivities of SAT (40%) and tardus-parvus wave patterns (60%) for vascular complications were not as substantial as the 81% sensitivity of SAT for HAS reported by Platt et al. with postoperative sonography [26]. It is noteworthy that these studies had different endpoints, HAT versus the composite of HAS or HAT.

Intraoperative Doppler ultrasound has clinical impact in identifying sequelae of the hepatic artery buffer response. Blood flows into the liver through the portal vein and hepatic artery. The liver receives nearly 25% of cardiac output and performs first-pass filtration from the splanchnic circulation [32,33]. The portal vein has a low pressure/low resistance circuit with higher blood inflow than the hepatic artery with high pressure and resistance but with lower blood inflow. The hepatic artery exhibits intrinsic regulation with compensatory changes to portal venous flow, known as the hepatic artery buffer response (HABR) [32]. Changes in arterial caliber to varied portal blood flow keep steady blood flow and ensure hepatic clearance with adequate oxygenation. This action is mediated by adenosine from intravascular ATP breakdown [32]. This phenomenon was previously attributed to steal effect [34]. Volume flowmetry in the portal vein and hepatic artery can be measured intraoperatively using doppler ultrasound during liver transplantation. Increased HABR is seen with lower graft to recipient liver volume ratio due to the concurrent portal vein hyperperfusion with a smaller graft. This increased flow can result in compensatory hepatic arterial hypoperfusion. The knowledge of such response is critical to help guide inflow modifications to maintain the excellent portal and arterial flow, especially in partial allografts. Thus, ultrasound surveillance at this vital point is recommended to prevent irreversible changes from ischemia, thrombosis, or cholestasis. 

Hepatic venous outflow obstruction occurs in 1-6% of OLTs due to inferior vena cava (IVC) torsion, compression, or anastomotic stenosis [34]. Typically, IVC stents manage long-term stenosis after OLT. Morochnik et al. describe the successful placement of IVC stent after detection of diminished intrahepatic blood flow during intraoperative DUS [32]. The early detection and intervention likely saved the graft. Hepatic venous outflow obstructions also occur in cases of hypercoagulability and autoimmune conditions [34]. When needed, close monitoring with DUS and IVC stenting improves vessel patency and decreases a patient’s risk for further complications. Portal vein (PV) complications are much less common than arterial complications. Portal vein thrombosis is 1-3% and typically occurs around one month after transplantation [35]. DUS findings would show a filling defect and decreased flow through the portal vein. Cheng et al. later reported that absent flow in the portal vein was associated with a prominent hepatic artery with an increased diameter and a hepatic artery RI < 0.5 in 73 pediatric patients undergoing LDLT indicative of portal vein thrombosis (PVT) [36]. Portal vein stenosis was less common than portal vein thrombosis. It is a later complication, presenting six months after transplantation, due to neointimal hyperplasia [36]. Stine et al. noted that less than 0.1% of their study population had preoperative PVT [37]. However, preoperative PVT was a significant risk factor for postoperative complications such as HATs, particularly in a high-risk donor liver [37-40]. However, it is a late and insidious complication and is not typically screened for in the perioperative to postoperative period. It is thus out of the scope of this review.

Alternative ultrasound types in transplant

Hom et al. performed a study comparing patients who had undergone a liver transplant with contrast-enhanced ultrasound to conventional color DUS [41]. Contrast-enhanced ultrasound improved flow visualization of the hepatic artery and portal vein, decreased scanning time, and differentiated between HAT and a patent artery when conventional DUS could not. Hom et al. also reported 100% specificity and sensitivity in contrast-enhanced ultrasound, compared to 100% and 91% for conventional DUS [41]. In another study by Wang et al., portal flow measured by transit time US (TTUS) and conventional DUS were compared, showing widely variable results between the two methods [42]. These studies indicate that type of US utilized may be pertinent when diagnosing vascular complications. Table 1 summarizes a review of the literature on postoperative and intraoperative Doppler ultrasonography in liver transplants. More investigation into specific ultrasound methods is needed to determine which is most efficacious in specific settings and set a path for more standardization of methods among centers.

**Table 1:** Summary of literature review for postoperative and intraoperative Doppler ultrasound in liver transplants DUS: Doppler ultrasonography; US: ultrasonography; LT: liver transplant; LDLT: living donor liver transplant; DDLT: deceased donor liver transplant; OLT: orthotopic liver transplant; SLT: segmental liver transplant; PVT: portal vein thrombosis; RI: resistive index; HAS: hepatic artery stenosis; HAT: hepatic artery thrombosis; eHAT: early hepatic artery thrombosis; SAT: systolic acceleration time; PSV: peak systolic velocity; GI: gastrointestinal; HA: hepatic artery; HARI: hepatic artery resistive index; PV: portal vein; Sn: sensitivity; Sp: specificity; AS: anastomotic biliary stricture; NAS: non-anastomotic biliary stricture; CTA: computed tomography angiography; GDA: gastroduodenal artery; IHARI: intrahepatic hepatic artery resistive index; IHASAT: intrahepatic hepatic artery systolic acceleration time; HRD: high-risk donor

Author	Background	Design	Result	Conclusion
Abdelaziz and Attia, 2016 [40]	Discussion of the role of intraoperative and postoperative DUS in LDLT	Literature review specifically in DUS use in LDLT	DUS is a noninvasive, inexpensive, and effective way to identify an array of vascular complications	DUS is a versatile tool for managing LDLTs in the operative and in the post-operative course
Cheng et al., 1998 [30]	Determine utility in using intraoperative DUS to detect vascular complications in LDLT	Prospective cohort study of 19 pediatric and 5 adult LTs who were assessed with intraoperative DUS	9 patients had vascular abnormalities recognized by intraoperative DUS with surgical correction and 100% graft survival	Use of intraoperative DUS allowed for early recognition and treatment of vascular complications and improved patient outcomes
Cheng et al., 2004 [36]	Assessment on the use of pre- and intraoperative DUS to detect PVT in pediatric LDLT	Retrospective cohort of 73 pediatric patients undergoing LDLT from 1994-2002	In patients with PVT, doppler flow was absent in portal vein when hepatic artery RI < 0.5	DUS is essential for detection of portal vein complications in LDLT
Choi et al., 2007 [13]	To determine the predictive benefit of intraoperative DUS for vascular complications compared against angiography after LDLT	Retrospective cohort study of 81 SLTs who underwent intraoperative DUS	The sensitivity, specificity, and negative predictive value (NPV) for HAS were 60.0%, 73.7%, and 84.9%, respectively, for tardus-parvus pattern and 40.0%, 83.6%, and 80.9%, respectively, for delayed SAT	Increased SAT of the hepatic artery, loss of triphasic hepatic vein waveform and a tardus-parvus pattern are predictive of vascular complications after transplant
Dodd et al., 1994 [17]	Assess value of hepatic RI and SAT in detecting HAS and HAT in LT patients postoperatively	Retrospective cohort of 125 LT patients	RI < 0.5 and SAT > 0.01sec significant for HAS or HAT. RI and SAT combined were predictive. PSV and absent HA flow were not predictive.	Diagnostic value of decreased RI and increased SAT in detecting complications of hepatic artery after LT
El-Nakeep and Ziska, 2022 [16]	Discussion of fundamentals, indications, and limitations of liver DUS	Review	DUS vessel assessment affected by excess probe pressure, the respiratory cycle, and GI transit	Limited assessment in patients who are obese, cannot control breathing, or who have not fasted for 4-6 hours
Garcia-Criado et al., 2003 [12]	Evaluation of the significance of HARI in immediate postoperative period of OLTs	Retrospective study of 90 patients who received DUS evaluation within 3 days of LT	46% of OLT patients had elevated HARIs within 72 hours of transplant. This was not associated with subsequent complications or morbidity/mortality at 5 years	HARI > 0.8 within the first 72 hours of OLT is not predictive of short-term graft complications or long-term graft function
Garcia-Criado et al., 2009 [22]	Description of normal and abnormal DUS waveforms in the hepatic artery following LT	Review	HAT is defined by absent DUS signal at liver hilum, arterial-steal syndrome shows absence of diastolic peaks with decreased PSV of HA	Vascular complications can be identified by their unique sonographic patterns before they present clinically
Gu et al., 2012 [31]	Comparison of intraoperative DUS findings between pediatric segmental LT patients with subsequent eHAT and those without	Pediatric segmental LTs were performed in 49 consecutive patients from 2006 to 2010	7 of 49 pediatric patients experienced eHAT, which was associated with decreased HA diameter, PSV, and RI	Determined HA diameter <2mm, PSV <40cm/s, RI <0.6 to be predictive of eHAT
Kimura et al., 2020 [11]	Exploration of different imaging to detect vascular and biliary complications of OLT	Review	DUS detects abnormalities in RI, diastolic flow, and waveforms when complications are present following LT	DUS is the first line imaging study to detect postoperative LT complications, followed by angiography
Lall et al., 2014 [27]	Reviewing normal postprocedural ultrasound findings after stenting for HAS	Retrospective cohort of 23 OLT patients who experienced HAS evaluated for changes in PSV, RI, and tardus-parvus waveforms by interval DUS screening	Pre-stenting RI below 0.40 had a strong correlation with restenosis. PSV above 300 cm/s after 90 days and RI below 0.55 after 3 days had a strong correlation with restenosis	DUS is a great screening test for restenosis after HAS following DDLT. Pre-stenting RI and post-stenting RI and PSV can have value in predicting restenosis days to months after intervention
Liao et al., 2021 [29]	Investigate utility of US in identifying aAS and NAS post-LT	Retrospective cohort of 1259 OLT patients, postoperative US referenced against cholangiography	NAS occurred later than AS, on average. NAS is associated with diminished or absent hilar bile duct lumen (Sn= 94%, Sp= 84%). AS identified by irregular intrahepatic duct dilatation	US is a reliable post-operative screening tool for both AS and NAS.
Mohamed et al., 2021 [28]	Evaluate diagnostic value of DUS in HAS detection compared to CTA	Retrospective cohort of 50 LDLT and DDLT recipients from 2005 to 2017	HAS identified in 9 (18%) patients. Intra- and extra-hepatic HA PSV was not a strong predictor. Intra-hepatic HARI < 0.585 (Sn= 87%, Sp=85%) and SAT > 0.045s (Sn=80%, Sp=91%).	RI < 0.585 and SAT >0.045s of intrahepatic HA strong predictors of HAS post-LT. When combined, IHARI and IHSAT have Sn=93% and Sp=88%. No reported time from transplant to HAS in this study.
Morochnik et al., 2021 [32]	Report of intraoperative hepatic venous outflow obstruction that was position dependent.	Case report	Labile hepatic venous outflow patency identified intraoperatively with observed congestion and diminished intraoperative DUS signal. Treated immediately with IVC stenting	Demonstration of qualitative intraoperative DUS parameter to aid in detection of immediate hepatic outflow complication in OLT
Nishida et al., 2005 [35]	GDA steal during LT detected by intraoperative DUS	Case report	DUS detected poor hepatic artery flow that improved following ligation of GDA	Intraoperative DUS is effective at diagnosing arterial steal syndrome
Nolten and Sproat, 1996 [21]	Evaluation of the time interval between US findings and definitive diagnosis of HAT after LT	Retrospective cohort of 202 liver transplant patients, DUS compared to angiography, surgery, autopsy	Sn for HAT 30 days before diagnosis was only 54%, compared to 82% on day of findings. Sp constant ~85%	DUS is a good screen, but angiography is recommended as Sn improves with clinical picture clarity
Platt et al., 1997 [26]	The use of doppler waveform analysis in detection of hepatic artery stenosis (HAS)	Spectral Doppler with arteriography charts reviewed for waveform, RI, SAT to determine if duplex doppler is useful for HAS prediction	Abnormal values for either RI or SAT are 81% sensitive and 61% specific for HAS. Abnormal values for both RI and SAT are 67% sensitive and 96% specific for HAS.	Abnormal values for both RI and SAT are a more accurate predictor of HAS than either only when doppler waveform analysis is used in HAS detection
Sanyal et al., 2014 [8]	Characterize the normal DUS changes seen postoperatively after LT and differentiate them from changes concerning for complications	Review	PSV may be variable in the postoperative period in normal LT. Decreased RI <0.55 should be concerning rather than an increased RI	Indirect DUS findings for HAS are predictive with Sn= 70-83% and Sp= 60-73%, indicating a satisfactory screening method
Stine et al., 2016 [37]	LT recipients with pre-transplant PVT receiving organs from high-risk donors (HRD) are at an increased risk of HAT.	Retrospective cross-sectional study of 60,404 liver transplant recipients above 18 between 2002 and 2015	A DRI cutoff of greater than 1.7 defined HRD. Following multivariable analysis, PVT with an HRD organ was the most significant independent risk factor for the development of HAT.	Patients with pre-transplant PVT who receive an organ from an HRD are at the highest risk for postoperative HAT
Stine et al., 2016 [38]	LT recipients with pre-transplant PVT are at increased risk for HAT	Retrospective study of 63,182 liver transplant patients above 18 from 2002 to 2014	PVT and donor risk index are associated with an increased independent risk of HAT	Pre-transplant PVT is independently associated with post-transplant HAT
Tezcan et al., 2022 [14]	PV flow shows no alterations to establish adequate blood supply in response to HA occlusion	Retrospective cohort of 33 adult patients with eHAT comparing PV velocity on DUS before and after HAT treatment	PV velocity was significantly decreased within 1-hour HAT treatment and confirmed resolution	Demonstrates a compensatory PV flow change in response to HA patency
Uzochukwu et. al., 2005 [23]	Evaluation of diagnostic value of postop DUS in vascular and biliary complications after OLTs	Cohort of 110 OLT patients with DUS investigation of main, left, and right HAs within 24-48hrs of LT	HARI < 0.6 in early postoperative period was associated with more graft complications overall	Low HARIs, rather high HARIs, in the early postoperative period are concerning for future complications
Vit et al., 2003 [15]	Analysis of qualitative and quantitative postoperative DUS findings predictive for HAS and HAT in adult OLT patients	Retrospective cohort of 136 adult OLT patients with postoperative DUS screening for HAS/HAT, confirmed with CTA	In 25 patients, 18.4% patients met DUS criteria for complication. Tardus-parvus waveform has the highest Sn and Sp for HAS or HAT. RI <0.5 and SAT>0.08 sec are specific but not sensitive	Decreased HARI <0.5 and PSV> 200cm/sec were not predictive of HAS and HAT. SAT> 0.08sec was a strong quantitative predictor but not as accurate as qualitative assessment of the tardus-parvus waveform in HA.
Wang et al., 2018 [42]	Assess agreement of transit time US and DUS for portal flow in LDLT (mean, median, and range)	Correlation study using Bland-Altman plot	Moderate agreement but with a wide range of variation	PV flow data between DUS and transit time US is not interchangeable

## Conclusions

The literature on intraoperative DUS in liver transplants is relatively sparse despite increased use. The present case reports and case series have demonstrated the utility of qualitative DUS assessment to detect immediate graft complications. However, only a handful of single-center studies have investigated quantitative parameters that would allow for increased standardization of DUS interpretation. This review concludes that focused investigations into intraoperative DUS findings are necessary for continued liver transplant success.
